# An Updated genome annotation for the model marine bacterium *Ruegeria pomeroyi* DSS-3

**DOI:** 10.1186/1944-3277-9-11

**Published:** 2014-12-08

**Authors:** Adam R Rivers, Christa B Smith, Mary Ann Moran

**Affiliations:** 1 Department of Marine Sciences, University of Georgia, Athens, GA, USA

**Keywords:** *Roseobacter*, DMSP

## Abstract

When the genome of *Ruegeria pomeroyi* DSS-3 was published in 2004, it represented the first sequence from a heterotrophic marine bacterium. Over the last ten years, the strain has become a valuable model for understanding the cycling of sulfur and carbon in the ocean. To ensure that this genome remains useful, we have updated 69 genes to incorporate functional annotations based on new experimental data, and improved the identification of 120 protein-coding regions based on proteomic and transcriptomic data. We review the progress made in understanding the biology of *R. pomeroyi* DSS-3 and list the changes made to the genome.

## Introduction

*Ruegeria pomeroyi* DSS-3 is an important model organism in studies of the physiology and ecology of marine bacteria [1]. It is a genetically tractable strain that has been essential for elucidating bacterial roles in the marine sulfur and carbon cycles [2,3] and the biology and genomics of the marine *Roseobacter* clade [4], a group that makes up 5–20% of bacteria in ocean surface waters [5,6]. Here we update the *R. pomeroyi* DSS-3 genome with 189 changes collected from the work of several research groups over the last ten years.

## Organism information

*Ruegeria pomeroyi* DSS-3 (formerly *Silicibacter pomeroyi* DSS-3 [7]) is a motile gram-negative alphaproteobacterium in the marine *Roseobacter* lineage [8]. This mesophilic, heterotrophic bacterium was isolated from an estuary in coastal Georgia, U.S.A [9] (Table 1).

**Table 1:** **Classification and general features of***Ruegeria pomeroyi***DSS-3 according to MIGS recommendations**[9]

**MIGS ID**	**Property**	**Term**	**Evidence code**^**a**^
	Current classification	Domain *Bacteria*	TAS [10]
		Phylum *Proteobacteria*	TAS [11]
		Class *Alphaproteobacteria*	TAS [12,13]
		Order *Rhodobacterales*	TAS [12,14]
		Family *Rhodobacteraceae*	TAS [12,15]
		Genus *Ruegeria*	TAS [7,16-18]
		Species *Ruegeria pomeroyi*	TAS [7,19]
		Type strain DSS-3 = ATCC 700808 T = DSM 15171	
	Gram stain	Negative	TAS [8]
	Cell shape	Rod	TAS [8]
	Motility	Motile	TAS [8]
	Sporulation	Non-sporulating	NAS
	Temperature range	Mesophile (10°C-40°C)	TAS [8]
	Optimum temperature	30°C	
	Carbon source	Acetate, ethanol, DL-β-hydroxybutyrate, glucose, succinate, acrylic acid, cysteic acid, glycerol, citrate, pyruvate, casamino acids, L-alanine, L-arginine, L-serine, L-taurine, L-methionine, DMSP and glycine betaine	TAS [8]
	Energy source	Carbon compounds	
	Terminal electron receptor	Oxygen	
MIGS-6	Habitat	Marine	
MIGS-6.3	Salinity	Optimum 100–400 mM	TAS [8]
MIGS-22	Oxygen	Aerobic	TAS [8]
MIGS-15	Biotic relationship	Free-living	TAS [8]
MIGS-14	Pathogenicity	Non-pathogenic	NAS
MIGS-4	Geographic location	Coastal Georgia, USA	TAS [8]
MIGS-5	Sample collection time	June 1996	NAS
MIGS-4.1 MIGS-4.2	Latitude – Longitude	31.989616 N, 81.022768 W	NAS
MIGS-4.3	Depth	Surface	NAS
MIGS-4.4	Altitude	Sea level	NAS

^a^Evidence codes - TAS: Traceable Author Statement (i.e., a direct report exists in the literature); NAS: Non-traceable Author Statement (i.e., not directly observed for the living, isolated sample, but based on a generally accepted property for the species, or anecdotal evidence).

## Genome sequencing information

### Genome project history

The genome of *R. pomeroyi* DSS-3 was sequenced in 2003 by The Institute for Genomic Research (now the J. Craig Venter Institute) using Sanger sequencing (Table 2), and was annotated using Glimmer 2 [20] and the TIGR Assembler [21]. The genome was published in 2004 [1].

**Table 2:** Project information

**MIGS ID**	**Property**	**Term**
MIGS-31	Finishing quality	Closed genome [1]
MIGS-28	Libraries used	1–2 kb and 12–15 kb inserts [1]
MIGS-29	Sequencing platforms	Sanger
MIGS-31.2	Fold coverage	Not reported
MIGS-30	Assemblers	The TIGR Assembler [20]
MIGS-32	Gene calling method	Glimmer 2.0 [20]
	Genome Database release	NCBI Refseq, release version #8
	Genbank ID	CP000031.2, CP000032.1
	Genbank Date of Release	December 16, 2004
	GOLD ID	Gc00242
	Project relevance	The first heterotrophic marine bacterium sequenced

## Genome properties

The *R. pomeroyi* DSS-3 genome contains a 4,109,437 bp circular chromosome (5 bp shorter than previously reported [1]) and a 491,611 bp circular megaplasmid, with a G + C content of 64.1 (Table 3). A detailed description of the genome is found in the original article [1].

**Table 3:** Genome statistics

**Attribute**	**Value**	**% of total**^**a**^
Genome size (bp)	4,601,048	100.0
DNA coding region (bp)	4,144,250	90.1
DNA G + C content (bp)	2,947,874	64.1
Total genes^b^	4371	100.0
RNA genes	64	1.5
Protein-coding genes	4276	97.8

^a^The total is based on either the size of the genome in base pairs or the total number of genes in the annotated genome.

^b^Also includes pseudogenes.

## Reannotation

The *R. pomeroyi* DSS-3 genome has been instrumental in expanding knowledge of the marine sulfur cycle, particularly the role of marine bacteria in controlling the flux of volatile sulfur to the atmosphere [3,22] and the bacterial transformations of dimethylsulfoniopropionate (DMSP) [3,23], dimethylsulfide, and sulfonates [24,25]. Since 2006, many of the genes mediating the uptake and metabolism of DMSP have been identified from the *R. pomeroyi* DSS-3 genome. These include the demethylation pathway genes *dmdABCD*[2,22] and the cleavage pathway genes *dddD, dddP, dddQ, dddW, acuK, acuN, dddA* and *dddC*[23,26,27]. Although many genes were identified first in *R. pomeroyi* DSS-3, these are now known to be widespread in ocean surface waters and harbored by a number of other major marine bacterial taxa [28]. *R. pomeroyi* DSS-3 also transforms sulfonates and has served as a model for identifying genes required for the degradation of 2,3-dihydroxypropane-1-sufonate (*hpsNOP*) [29], L-cysteate (*cuyARZ*) [30], taurine (*tauXY*) and n-acetyltaurine (*naaST*) [24,31,32], 3-sulfolactate (*slcD, suyAB*) [29,33] and isethionate (*iseJ*) [25].

Members of the marine *Roseobacter* lineage are capable of oxidizing sulfite and thiosulfate [34,35], and the genome sequence of *R. pomeroyi* DSS-3 revealed the *sox* gene cluster that mediates these processes [1,4]. Recently, the reverse dissimilatory sulfite reductase gene cluster was found in sediment-dwelling roseobacters, and homologs to the sulfite reductase genes from this pathway (*soeABC*) were identified in the *R. pomeroyi* DSS-3 genome [36]. *R. Pomeroyi* DSS-3 was initially studied as a member of an ecologically important bacterial taxon that appeared unusually amenable to cultivation [5], but has now played a major role in improving our understanding of global sulfur transformations.

Studies of the *R. pomeroyi* DSS-3 genome have also provided a better understanding of the genes involved in processing organic nitrogen compounds, such as taurine and N-acetyltaurine [24,31,32]. The bacterium can catabolize lysine by using the saccharopine pathway, which is used by many plants and animals, or by using the lysine dehydrogenase pathway. Under high salt conditions, it preferentially uses the latter pathway, leading to biosynthesis of the osmolyte aminoadipate. The function of several genes in both lysine pathways has recently been experimentally verified [37].

*R. pomeroyi* DSS-3 genome hosts at least 28 tripartite ATP-independent periplasmic (TRAP) transporters [1]. While the substrates for many of these transporters are not yet known, the TRAP transporters responsible for the uptake of 2,3-dihydroxypropane-1-sufonate (*hpsKLM*) [29], isethionate (*iseKLM*) [25], and ectoine and hydroxyectoine have been characterized (*uehABC*) [38,39]. Ectoine and hydroxyectoine are used as compatible solutes by some bacteria and phytoplankton, although *R. pomeroyi* DSS-3 can also assimilate carbon and nitrogen from them [39]. Several genes involved in ectoine metabolism (*doe, eut, ueh*) have been found in the *R. pomeroyi* DSS-3 genome based on homology with genes in *Halomonas elongata* DSM 2581 T [40].

Progress has been made in understanding the mechanisms of metal uptake in *R. pomeroyi* DSS-3. The manganese uptake regulator *mur* has been experimentally validated, as have the ABC transporter genes for manganese metabolism (*sitABCD*) [41]. In total, 69 annotation changes were made based on new experimental data identifying genes responsible for carbon, nitrogen, sulfur, and metal uptake and metabolism [42].

Proteomics [42] and mRNA sequencing have resulted in 120 protein coding regions being identified, removed or corrected in the updated genome. A detailed proteomic study of *R. pomeroyi* DSS-3 under diverse growth conditions resulted in the identification of 26 novel open reading frames (ORFs) and 5 sequencing errors [42]. The function of most of the new genes is not known and 16 of the expressed polypeptides do not have known homologs. The 26 ORFs missed in the original annotation is a significant number but less than the 1% error rate predicted for Glimmer 2 [20]. The proteomic analysis was also able to correct the start sites of 64 genes [42], enhancing the information that had been obtained only from the DNA sequence [20]. Many of the ORFs identified by proteomics were independently confirmed using strand-specific messenger RNA sequences from continuous cultures [43] and the gene calling software Glimmer 3 [44]. This method also identified several genes that were originally annotated in the wrong orientation, including a novel bacterial collagen gene (SPO1999).

A list of genome updates based on these biochemical, genetic, and -omics approaches is provided in Table 4, and full details in Additional file 1: Table S1. The updated annotations have been incorporated into the official genome record at the National Center for Biotechnology Information (Bethesda, MD, USA) under accession numbers CP000031.2 and CP000032.1 and Roseobase (http://roseobase.org).

**Table 4:** Updates and corrections to the genome sequence

**Accession**	**Gene locus**	**CDS**	**Gene**	**Type of change**
YP_166946	SPO1707a	Branched-chain amino acid ABC transporter, ATP-binding protein, putative		Locus name
YP_167418	SPO2192a	N-formylglutamate amidohydrolase	*hutG*	Locus name
YP_165298	SPO0025	Hydrolase, NUDIX family		ORF position
YP_165304	SPO0031	ErfK/YbiS/YcfS/YnhG family protein		ORF position
YP_165330	SPO0056	Hypothetical protein		ORF position
YP_165481	SPO0212	Hypothetical protein		ORF position
YP_165606	SPO0343	2-oxoglutarate dehydrogenase, E2 component, dihydrolipoamide succinyltransferase	*sucB*	ORF position
YP_165666	SPO0403	Conserved domain protein		ORF position
YP_165678	SPO0415	D-isomer specific 2-hydroxyacid dehydrogenase family protein		ORF position
YP_165703	SPO0440	Thioesterase family protein		ORF position
YP_165709	SPO0446	ABC transporter, ATP-binding protein		ORF position
YP_165719	SPO0456	Hypothetical protein		ORF position
YP_165753	SPO0491	Hypothetical protein		ORF position
YP_165766	SPO0504	Hypothetical protein		ORF position
YP_165767	SPO0505	Ribosomal protein L15	*rplO*	ORF position
YP_165860	SPO0600	Carboxynorspermidine decarboxylase	*nspC*	ORF position
YP_165899	SPO0644	Hypothetical protein		ORF position
YP_165937	SPO0682	Monooxygenase family protein		ORF position
YP_165950	SPO0695	Hypothetical protein		ORF position
YP_008877643	SPO0876a	Hypothetical protein		ORF position
YP_166130	SPO0877	Conserved domain protein		ORF position
YP_166199	SPO0946	Phosphomannomutase/phosphoglucomutase	*algC*	ORF position
YP_166255	SPO1003	ATP-dependent Clp protease, proteolytic subunit ClpP	*clpP*	ORF position
YP_166256	SPO1004	ATP-dependent Clp protease, ATP-binding subunit ClpX	*clpX*	ORF position
YP_166357	SPO1106	Hypothetical protein		ORF position
YP_166419	SPO1172	FMN-dependent alpha-hydroxy acid dehydrogenase family protein		ORF position
YP_166421	SPO1174	DNA helicase II, putative		ORF position
YP_166518	SPO1273	Thymidylate synthase, flavin-dependent	*thyX*	ORF position
YP_166577	SPO1334	Hypothetical protein		ORF position
YP_166601	SPO1359	Pyruvate, phosphate dikinase	*ppdK*	ORF position
YP_166628	SPO1386	HIT family protein		ORF position
YP_166803	SPO1562	Glycine cleavage system T protein, putative		ORF position
YP_166874	SPO1633	Hypothetical protein		ORF position
YP_167013	SPO1776	Pyridine nucleotide-disulphide oxidoreductase family protein		ORF position
YP_167049	SPO1812	Adenylate kinase	*adk-2*	ORF position
YP_167155	SPO1920	Tellurite resistance protein	*trgB*	ORF position
YP_167190	SPO1955	Glutaryl-CoA dehydrogenase	*gcdH*	ORF position
YP_167207	SPO1972	Nodulation protein N		ORF position
YP_167208	SPO1973	3-dehydroquinte dehydratase, type II	*aroQ*	ORF position
YP_167281	SPO2051	DNA gyrase, A subunit	*gyrA*	ORF position
YP_167284	SPO2054	Cytochrome c oxidase assembly protein		ORF position
YP_167368	SPO2141	Pyridoxamine 5''-phosphate oxidase, putative		ORF position
YP_167443	SPO2217	Excinuclease		ORF position
YP_167514	SPO2290	Hypothetical protein		ORF position
YP_167549	SPO2326	Hypothetical protein		ORF position
YP_167562	SPO2339	Enoyl-CoA hydratase/isomerase family protein		ORF position
YP_167570	SPO2347	Hypothetical protein		ORF position
YP_167571	SPO2348	Sarcosine oxidase, beta subunit family		ORF position
YP_167714	SPO2499	Hypothetical protein		ORF position
YP_167808	SPO2595	Hypothetical protein		ORF position
YP_167819	SPO2608	Aldehyde dehydrogenase, putative		ORF position
YP_167822	SPO2612	DNA-binding protein HU, putative		ORF position
YP_008877659	SPO2723a	Hypothetical protein		ORF position
YP_167934	SPO2724	Hypothetical protein		ORF position
YP_167992	SPO2785	NADH dehydrogense I, B subunit	*nuoB*	ORF position
YP_168024	SPO2816	Peptide/nickel/opine uptake family ABC transporter, permease protein		ORF position
YP_168061	SPO2853	Cobalt chelatase, CobS subunit		ORF position
YP_168080	SPO2872	Cobyrinic acid a,c-diamide synthase	*cobB*	ORF position
YP_168096	SPO2888	Membrane protein, putative		ORF position
YP_168125	SPO2917	Glutathione S-transferase family protein		ORF position
YP_168133	SPO2925	Sporulation related		ORF position
YP_168143	SPO2936	Hypothetical protein		ORF position
YP_168150	SPO2942	Hypothetical protein		ORF position
YP_168197	SPO2991	Nitroreductase family protein		ORF position
YP_168209	SPO3003	AMP-binding enzyme		ORF position
YP_168292	SPO3089	ATPase, putative		ORF position
YP_168317	SPO3114	Hypothetical protein		ORF position
YP_168354	SPO3151	HAD-superfamily subfamily IIA hydrolase, TIGR01459		ORF position
YP_168406	SPO3203	Guanosine-3',5'-bis(Diphosphate) 3'-pyrophosphohydrolase, putative		ORF position
YP_168423	SPO3220	Aminotransferase, classes I and II		ORF position
YP_168448	SPO3245	Nicotinate-nucleotide pyrophosphorylase	*nadC*	ORF position
YP_168475	SPO3278	Orotidine 5'-phosphate decarboxylase	*pyrF*	ORF position
YP_168540	SPO3344	Cys/Met metabolism PLP-dependent enzyme family protein		ORF position
YP_168563	SPO3367	Deoxyribose-phosphate aldolase	*deoC*	ORF position
YP_168618	SPO3422	ATP-dependent protease La domain protein		ORF position
YP_168712	SPO3517	Preprotein translocase, SecE subunit	*secE*	ORF position
YP_168722	SPO3527	Universal stress protein family protein		ORF position
YP_168735	SPO3540	Hypothetical protein		ORF position
YP_168802	SPO3607	Hypothetical protein		ORF position
YP_168911	SPO3717	Cytosol aminopeptidase family protein		ORF position
YP_168940	SPO3746	Adenine deaminse	*ade*	ORF position
YP_169017	SPO3829	S-formylglutathione hydrolase, putative		ORF position
YP_169021	SPO3833	ATP-dependent RNA helicase, DEAD/DEAH box family		ORF position
YP_164889	SPOA0058	Glycine cleavage system protein H	*gcvH-2*	ORF position
YP_165979	SPO0725	Bacterial SH3 domain family protein		ORF position, Function
YP_167233	SPO1999	Collagen domain protein		ORF position, Function
YP_008877641	SPO0561	ABC transporter		Sequence
YP_008877654	SPO2024	Aminotransferase		Sequence
YP_008877662	SPO3316a	Stress protein		Sequence
YP_008877661	SPO3904	Heat shock protein		Sequence
YP_167141	SPO1905	Fumarate hydratase, class II	*fumC*	Sequence, ORF position
YP_165491	SPO0222	Alanine dehydrogenase	*ald*	Function
YP_165503	SPO0234	Lysine dehydrogenase	*lysdh*	Function
YP_165504	SPO0235	α-aminoadipic-δ-semialdehyde dehydrogenase	*aasadh*	Function
YP_165716	SPO0453	DMSP lyase	*dddW*	Function
YP_165850	SPO0590	LacI family transcriptional regulator	*hpsR*	Function
YP_165851	SPO0591	Dihydroxypropanesulfonate (DHPS) TRAP transporter	*hpsK*	Function
YP_165852	SPO0592	Dihydroxypropanesulfonate (DHPS) TRAP transporter	*hpsL*	Function
YP_165853	SPO0593	Dihydroxypropanesulfonate (DHPS) TRAP transporter	*hpsM*	Function
YP_165854	SPO0594	Dihydroxypropanesulfonate-3-dehydrogenase	*hpsN*	Function
YP_165855	SPO0595	R or S-dihydroxypropanesulfonate-2-dehydrogenase	*hpsO*	Function
YP_165856	SPO0596	S or R-dihydroxypropanesulfonate-2-dehydrogenase	*hpsP*	Function
YP_165857	SPO0597	UspA stress protein	*hpsQ*	Function
YP_165858	SPO0598	Membrane-bound sulfolactate dehydrogenase	*slcD*	Function
YP_165912	SPO0657	Metallochaperone, putative	*naaT*	Function
YP_165913	SPO0658	N-acetyltaurine amidohydrolase	*naaS*	Function
YP_165914	SPO0659	LysR family transcriptional regulator	*naaR*	Function
YP_165915	SPO0660	N-acetyltaurine ABC transporter, periplasmic binding protein	*naaA*	Function
YP_165916	SPO0661	N-acetyltaurine ABC transporter, permease protein	*naaB*	Function
YP_165917	SPO0662	N-acetyltaurine ABC transporter, permease protein	*naaB'*	Function
YP_165918	SPO0663	N-acetyltaurine ABC transporter, ATP-binding protein	*naaC*	Function
YP_165919	SPO0664	N-acetyltaurine ABC transporter, ATP-binding protein	*naaC'*	Function
YP_165928	SPO0673	Taurine-pyruvate aminotransferase	*tpa*	Function
YP_165929	SPO0674	Taurine ABC transporter, periplasmic taurine-binding protein	*tauA*	Function
YP_165930	SPO0675	Taurine ABC transporter, ATP-binding protein	*tauB*	Function
YP_165931	SPO0676	Taurine ABC transporter, permease protein	*tauC*	Function
YP_166034	SPO0781	Phosphonate ABC transporter substrate-binding protein	*phnD*	Function
YP_166387	SPO1136	Diaminobutyric acid transaminase	*doeD*	Function
YP_166388	SPO1137	Aspartate-semialdehyde dehydrogenase	*doeC*	Function
YP_166389	SPO1138	AsnC/Lrp-like DNA-binding protein, transcriptional regulator	*doeX*	Function
YP_166390	SPO1139	Nα-acetyl-L-2,4-diaminobutyric acid deacetylase	*doeB*	Function
YP_166391	SPO1140	Ectoine hydrolase	*doeA*	Function
YP_166392	SPO1141	Ectoine utilization protein EutC	*eutC*	Function
YP_166394	SPO1143	Ectoine utilization protein EutA	*eutA*	Function
YP_166396	SPO1145	Ectoine/5-hydroxyectoine TRAP transporter, periplasmic binding protein	*uehC*	Function
YP_166397	SPO1146	Ectoine/5-hydroxyectoine TRAP transporter, small integral membrane protein	*uehB*	Function
YP_166398	SPO1147	Ectoine/5-hydroxyectoine TRAP transporter, large integral membrane protein	*uehA*	Function
YP_166399	SPO1148	Transcriptional regulator, GntR family	*gntR*	Function
YP_166792	SPO1551	Trimethylamine (TMA) monooxygenase	*tmm*	Function
YP_166837	SPO1596	DMSP lyase	*dddQ*	Function
YP_166942	SPO1703	DMSP lyase	*dddD*	Function
YP_167149	SPO1914	NADPH-dependent acrylyl-CoA reductase	*acuI*	Function
YP_167183	SPO1948	Phosphate ABC transporter substrate-binding protein	*pstS*	Function
YP_167275	SPO2045	3-methylmercaptopropionyl-CoA ligase	*dmdB*	Function
YP_167522	SPO2299	DMSP lyase	*dddP*	Function
YP_167578	SPO2355	Isethionate dissimilation regulator	*iseR*	Function
YP_167579	SPO2356	Isethionate TRAP transporter	*iseM*	Function
YP_167580	SPO2357	Isethionate TRAP transporter	*iseL*	Function
YP_167581	SPO2358	Isethionate TRAP transporter	*iseK*	Function
YP_167582	SPO2359	Isethionate dehydrogenase	*iseJ*	Function
YP_167694	SPO2477	Manganese uptake regulator	*mur*	Function
YP_168390	SPO3187	(2R)-3-sulfolactate dehydrogenase	*comC*	Function
YP_168503	SPO3307	Lysine-ketoglutarate reductase	*lkr*	Function
YP_168559	SPO3363	Manganese ABC transporter, permease protein	*sitD*	Function
YP_168560	SPO3364	Manganese ABC transporter, permease protein	*sitC*	Function
YP_168561	SPO3365	Manganese ABC transporter, ATP-binding protein	*sitB*	Function
YP_168562	SPO3366	Manganese ABC transporter, periplasmic protein	*sitA*	Function
YP_168752	SPO3557	Sulfite dehydrogenase subunit SoeC; transmembrane sulfate transporter	*soeC*	Function
YP_168753	SPO3558	Sulfite dehydrogenase iron-sulfur cluster-binding subunit SoeB; cytosolic protein	*soeB*	Function
YP_168754	SPO3559	Sulfite dehydrogenase molybdopterin cofactor-binding subunit SoeA; cytosolic protein	*soeA*	Function
YP_168755	SPO3560	Phosphate acetyltransferase	*pta*	Function
YP_168757	SPO3562	Taurine transcriptional regulator	*tauR*	Function
YP_168992	SPO3804	3-methylmercaptopropionyl-CoA dehydrogenase	*dmdC*	Function
YP_168993	SPO3805	Methylthioacryloyl-CoA hydratase	*dmdD*	Function
YP_164988	SPOA0157	Sulfite exporter	*cuyZ*	Function
YP_164989	SPOA0158	L-cysteate sulfo-lyase	*cuyA*	Function
YP_164990	SPOA0159	Transcriptional regulator cuyR	*cuyR*	Function
YP_165136	SPOA0309	Sulphoacetaldehyde acetyltransferase		Function
YP_008877636	SPO0344a	Hypothetical protein		New ORF
YP_008877637	SPO0346a	Hypothetical protein		New ORF
YP_008877638	SPO0360a	Hypothetical protein		New ORF
YP_008877639	SPO0491a	Hypothetical protein		New ORF
YP_008877640	SPO0504a	Hypothetical protein		New ORF
YP_008877642	SPO0628a	Hypothetical protein		New ORF
YP_008877644	SPO1044a	Hypothetical protein		New ORF
YP_008877645	SPO1094a	Hypothetical protein		New ORF
YP_008877646	SPO1226a	Hypothetical protein		New ORF
YP_008877647	SPO1252a	Transcriptional regulator		New ORF
YP_008877648	SPO1310a	Hypothetical protein		New ORF
YP_008877649	SPO1337a	Hypothetical protein		New ORF
YP_008877650	SPO1352a	Hypothetical protein		New ORF
YP_008877651	SPO1356a	Signal transduction		New ORF
YP_008877652	SPO1364a	Hypothetical protein		New ORF
YP_008877653	SPO1412a	Hypothetical protein		New ORF
YP_008877655	SPO2213a	Hypothetical protein		New ORF
YP_008877656	SPO2341a	Hypothetical protein		New ORF
YP_008877657	SPO2478	RNA helicase		New ORF
YP_008877658	SPO2652a	Polyketide cyclase		New ORF
YP_008877660	SPO2973a	Hypothetical protein		New ORF
YP_008877663	SPO3452a	Hypothetical protein		New ORF
YP_008877664	SPO3498a	Hypothetical protein		New ORF
YP_008877665	SPO3673a	Hypothetical protein		New ORF
AHC32567	SPOA0087a	Esterase-lipase		New ORF
AHC32568	SPOA0272a	Hypothetical protein		New ORF
YP_165305	-	Hypothetical protein		Removed ORF
YP_165605	-	Hypothetical protein		Removed ORF
YP_166669	-	Hypothetical protein		Removed ORF
YP_168865	-	Hypothetical protein		Removed ORF
YP_165238	-	Hypothetical protein		Removed ORF

## Conclusion

Ten years after the publication of the *Ruegeria pomeroyi* DSS-3 genome sequence, advances in knowledge of gene function and structural genome features motivated an annotation update. As an ecologically-relevant heterotrophic marine bacterium that is amenable to laboratory studies and genetic manipulation, *R. pomeroyi* is serving as a valuable model organism for investigations of the ecology, biochemistry, and biogeochemistry of ocean microbes.

## Competing interests

The authors declare that they have no competing interests.

## Authors’ contributions

ARR conceived of the study, carried out the bioinformatics analyses, and wrote the manuscript. CBS carried out the bioinformatics analyses and wrote the manuscript. MAM conceived of the study and wrote the manuscript. All authors read and approved the final manuscript.

## Supplementary Material

Additional file 1: Table S1 Full details of updates and corrections to the *Ruegeria pomeroyi* DSS-3 genome sequence. Click here for file
