# Erratum to: An updated genome annotation for the model marine bacterium *Ruegeria pomeroyi* DSS-3

**DOI:** 10.1186/s40793-015-0107-9

**Published:** 2015-11-26

**Authors:** Adam R. Rivers, Christa B. Smith, Mary Ann Moran

**Affiliations:** Department of Marine Sciences, University of Georgia, Athens, GA USA

After publication of this study [1], the authors noticed that the reference numbers given in the original version of the Additional file 1: Table S1 were incorrect. The corrected Additional file 1: Table S1 can be found below:

## Additional file

Additional file 1: Table S1. Full details of updates and corrections to the *Ruegeria pomeroyi* DSS-3 genome sequence. (DOCX 61 kb)
