# Supplementary material for: Erratum to: An updated genome annotation for the model marine bacterium Ruegeria pomeroyi DSS-3
Source: Stand Genomic Sci. 2015 Nov 26;10:112. doi: 10.1186/s40793-015-0107-9 (PMC4660654; doi:10.1186/s40793-015-0107-9)
Supplement: Additional file 1: Table S1. — Full details of updates and corrections to the Ruegeria pomeroyi DSS-3 genome sequence. (DOCX 61 kb) [file 40793_2015_107_MOESM1_ESM.docx]

**Table S1: Full details of updates and corrections to the *Ruegeria pomeroyi* DSS-3 genome sequence.**

| **Protein** | **Accession** | **Gene Locus** | **CDS** | **Gene** | **Type of change** | **Previous Locus** | **Note** | **Reference** |
| --- | --- | --- | --- | --- | --- | --- | --- | --- |
| AAV97140 | YP_166946 | SPO1707a | Branched-chain amino acid ABC transporter, ATP-binding protein, putative |  | Locus name | SPOA0447 | Initially assigned to the plasmid | This work |
| AAV97141 | YP_167418 | SPO2192a | N-formylglutamate amidohydrolase | *hutG* | Locus name | SPOA0448 | Initially assigned to the plasmid | This work |
| AAV93356 | YP_165298 | SPO0025 | Hydrolase, NUDIX family |  | ORF position |  |  | [42] |
| AAV93362 | YP_165304 | SPO0031 | ErfK/YbiS/YcfS/YnhG family protein |  | ORF position |  | Determined by RNAseq, Glimmer 3 | [43], This work |
| AAV93387 | YP_165330 | SPO0056 | Hypothetical protein |  | ORF position |  | Determined by RNAseq, Glimmer 3, ORF Finder | [43], This work |
| AAV93537 | YP_165481 | SPO0212 | Hypothetical protein |  | ORF position |  |  | [42] |
| AAV93661 | YP_165606 | SPO0343 | 2-oxoglutarate dehydrogenase, E2 component, dihydrolipoamide succinyltransferase | *sucB* | ORF position |  |  | [42] |
| AAV93721 | YP_165666 | SPO0403 | Conserved domain protein |  | ORF position |  |  | [42] |
| AAV93733 | YP_165678 | SPO0415 | D-isomer specific 2-hydroxyacid dehydrogenase family protein |  | ORF position |  |  | [42] |
| AAV93758 | YP_165703 | SPO0440 | Thioesterase family protein |  | ORF position |  |  | [42] |
| AAV93764 | YP_165709 | SPO0446 | ABC transporter, ATP-binding protein |  | ORF position |  |  | [42] |
| AAV93774 | YP_165719 | SPO0456 | Hypothetical protein |  | ORF position |  |  | [42] |
| AAV93808 | YP_165753 | SPO0491 | Hypothetical protein |  | ORF position |  |  | [42] |
| AAV93821 | YP_165766 | SPO0504 | Hypothetical protein |  | ORF position | SPO_PG027 |  | [42] |
| AAV93822 | YP_165767 | SPO0505 | Ribosomal protein L15 | *rplO* | ORF position |  |  | [42] |
| AAV93915 | YP_165860 | SPO0600 | Carboxynorspermidine decarboxylase | *nspC* | ORF position |  |  | [42] |
| AAV93952 | YP_165899 | SPO0644 | Hypothetical protein |  | ORF position |  |  | [42] |
| AAV93990 | YP_165937 | SPO0682 | Monooxygenase family protein |  | ORF position |  |  | [42] |
| AAV94002 | YP_165950 | SPO0695 | Hypothetical protein |  | ORF position |  |  | [42] |
| AHB86012 | YP_008877643 | SPO0876a | Hypothetical protein |  | ORF position | SPO_PG034 |  | [42] |
| AAV94182 | YP_166130 | SPO0877 | Conserved domain protein |  | ORF position |  |  | [42] |
| AAV94251 | YP_166199 | SPO0946 | Phosphomannomutase/phosphoglucomutase | *algC* | ORF position |  |  | [42] |
| AAV94307 | YP_166255 | SPO1003 | ATP-dependent Clp protease, proteolytic subunit ClpP | *clpP* | ORF position |  |  | [42] |
| AAV94308 | YP_166256 | SPO1004 | ATP-dependent Clp protease, ATP-binding subunit ClpX | *clpX* | ORF position |  |  | [42] |
| AAV94406 | YP_166357 | SPO1106 | Hypothetical protein |  | ORF position |  |  | [42] |
| AAV94468 | YP_166419 | SPO1172 | FMN-dependent alpha-hydroxy acid dehydrogenase family protein |  | ORF position |  | Confirmed by RNAseq | [42,43], This work |
| AAV94470 | YP_166421 | SPO1174 | DNA helicase II, putative |  | ORF position |  |  | [42] |
| AAV94565 | YP_166518 | SPO1273 | Thymidylate synthase, flavin-dependent | *thyX* | ORF position |  |  | [42] |
| AAV94623 | YP_166577 | SPO1334 | Hypothetical protein |  | ORF position |  |  | [42] |
| AAV94647 | YP_166601 | SPO1359 | Pyruvate, phosphate dikinase | *ppdK* | ORF position |  |  | [42] |
| AAV94674 | YP_166628 | SPO1386 | HIT family protein |  | ORF position |  |  | [42] |
| AAV94849 | YP_166803 | SPO1562 | Glycine cleavage system T protein, putative |  | ORF position |  |  | [42] |
| AAV94920 | YP_166874 | SPO1633 | Hypothetical protein |  | ORF position | SPO_PG030 |  | [42] |
| AAV95055 | YP_167013 | SPO1776 | Pyridine nucleotide-disulphide oxidoreductase family protein |  | ORF position |  |  | [42] |
| AAV95091 | YP_167049 | SPO1812 | Adenylate kinase | *adk-2* | ORF position |  |  | [42] |
| AAV95197 | YP_167155 | SPO1920 | Tellurite resistance protein | *trgB* | ORF position |  |  | [42] |
| AAV95231 | YP_167190 | SPO1955 | Glutaryl-CoA dehydrogenase | *gcdH* | ORF position |  |  | [42] |
| AAV95248 | YP_167207 | SPO1972 | Nodulation protein N |  | ORF position |  |  | [42] |
| AAV95249 | YP_167208 | SPO1973 | 3-dehydroquinte dehydratase, type II | *aroQ* | ORF position |  |  | [42] |
| AAV95322 | YP_167281 | SPO2051 | DNA gyrase, A subunit | *gyrA* | ORF position |  |  | [42] |
| AAV95325 | YP_167284 | SPO2054 | Cytochrome c oxidase assembly protein |  | ORF position |  |  | [42] |
| AAV95409 | YP_167368 | SPO2141 | pyridoxamine 5''-phosphate oxidase, putative |  | ORF position |  |  | [42] |
| AAV95483 | YP_167443 | SPO2217 | Excinuclease |  | ORF position | SPO_PG033 |  | [42] |
| AAV95554 | YP_167514 | SPO2290 | Hypothetical protein |  | ORF position | SPO_PG028 |  | [42] |
| AAV95588 | YP_167549 | SPO2326 | Hypothetical protein |  | ORF position |  |  | [42] |
| AAV95601 | YP_167562 | SPO2339 | Enoyl-CoA hydratase/isomerase family protein |  | ORF position |  |  | [42] |
| AAV95609 | YP_167570 | SPO2347 | Hypothetical protein |  | ORF position |  |  | [42] |
| AAV95610 | YP_167571 | SPO2348 | Sarcosine oxidase, beta subunit family |  | ORF position |  |  | [42] |
| AAV95749 | YP_167714 | SPO2499 | Hypothetical protein |  | ORF position |  |  | [42] |
| AAV95843 | YP_167808 | SPO2595 | Hypothetical protein |  | ORF position | SPO_PG035 |  | [42] |
| AAV95854 | YP_167819 | SPO2608 | Aldehyde dehydrogenase, putative |  | ORF position |  |  | [42] |
| AAV95857 | YP_167822 | SPO2612 | DNA-binding protein HU, putative |  | ORF position |  |  | [42] |
| AHB86028 | YP_008877659 | SPO2723a | Hypothetical protein |  | ORF position | SPO_PG031 |  | [42] |
| AAV95969 | YP_167934 | SPO2724 | Hypothetical protein |  | ORF position |  | Confirmed by RNAseq | [42,43], This work |
| AAV96026 | YP_167992 | SPO2785 | NADH dehydrogense I, B subunit | *nuoB* | ORF position |  |  | [42] |
| AAV96057 | YP_168024 | SPO2816 | Peptide/nickel/opine uptake family ABC transporter, permease protein |  | ORF position |  |  | [42] |
| AAV96094 | YP_168061 | SPO2853 | Cobalt chelatase, CobS subunit |  | ORF position |  |  | [42] |
| AAV96113 | YP_168080 | SPO2872 | Cobyrinic acid a,c-diamide synthase | *cobB* | ORF position |  |  | [42] |
| AAV96129 | YP_168096 | SPO2888 | Membrane protein, putative |  | ORF position |  |  | [42] |
| AAV96158 | YP_168125 | SPO2917 | Glutathione S-transferase family protein |  | ORF position |  |  | [42] |
| AAV96166 | YP_168133 | SPO2925 | Sporulation related |  | ORF position | SPO_PG029 |  | [42] |
| AAV96177 | YP_168143 | SPO2936 | Hypothetical protein |  | ORF position |  | Determined by Glimmer 3 | This work |
| AAV96183 | YP_168150 | SPO2942 | Hypothetical protein |  | ORF position |  |  | [42] |
| AAV96229 | YP_168197 | SPO2991 | Nitroreductase family protein |  | ORF position |  |  | [42] |
| AAV96241 | YP_168209 | SPO3003 | AMP-binding enzyme |  | ORF position |  |  | [42] |
| AAV96324 | YP_168292 | SPO3089 | ATPase, putative |  | ORF position |  |  | [42] |
| AAV96349 | YP_168317 | SPO3114 | Hypothetical protein |  | ORF position |  |  | [42] |
| AAV96386 | YP_168354 | SPO3151 | HAD-superfamily subfamily IIA hydrolase, TIGR01459 |  | ORF position |  |  | [42] |
| AAV96438 | YP_168406 | SPO3203 | Guanosine-3',5'-bis(Diphosphate) 3'-pyrophosphohydrolase, putative |  | ORF position |  |  | [42] |
| AAV96455 | YP_168423 | SPO3220 | Aminotransferase, classes I and II |  | ORF position |  |  | [42] |
| AAV96480 | YP_168448 | SPO3245 | Nicotinate-nucleotide pyrophosphorylase | *nadC* | ORF position |  |  | [42] |
| AAV96507 | YP_168475 | SPO3278 | Orotidine 5'-phosphate decarboxylase | *pyrF* | ORF position |  |  | [42] |
| AAV96570 | YP_168540 | SPO3344 | Cys/Met metabolism PLP-dependent enzyme family protein |  | ORF position |  |  | [42] |
| AAV96594 | YP_168563 | SPO3367 | Deoxyribose-phosphate aldolase | *deoC* | ORF position |  |  | [42] |
| AAV96648 | YP_168618 | SPO3422 | ATP-dependent protease La domain protein |  | ORF position |  |  | [42] |
| AAV96742 | YP_168712 | SPO3517 | Preprotein translocase, SecE subunit | *secE* | ORF position |  |  | [42] |
| AAV96752 | YP_168722 | SPO3527 | Universal stress protein family protein |  | ORF position |  |  | [42] |
| AAV96765 | YP_168735 | SPO3540 | Hypothetical protein |  | ORF position | SPO_PG032 |  | [42] |
| AAV96831 | YP_168802 | SPO3607 | Hypothetical protein |  | ORF position |  |  | [42] |
| AAV96938 | YP_168911 | SPO3717 | Cytosol aminopeptidase family protein |  | ORF position |  |  | [42] |
| AAV96967 | YP_168940 | SPO3746 | Adenine deaminse | *ade* | ORF position |  |  | [42] |
| AAV97043 | YP_169017 | SPO3829 | S-formylglutathione hydrolase, putative |  | ORF position |  |  | [42] |
| AAV97047 | YP_169021 | SPO3833 | ATP-dependent RNA helicase, DEAD/DEAH box family |  | ORF position |  |  | [42] |
| AAV97198 | YP_164889 | SPOA0058 | Glycine cleavage system protein H | *gcvH-2* | ORF position |  | Confirmed by RNAseq | [42,43], This work |
| AAV94031 | YP_165979 | SPO0725 | Bacterial SH3 domain family protein |  | ORF position, Function |  | Determined by RNAseq, Glimmer 3, ORF Finder | [43], This work |
| AAV95274 | YP_167233 | SPO1999 | Collagen domain protein |  | ORF position, Function |  | Determined by RNAseq, Glimmer 3, ORF Finder | [43], This work |
| AHB86010 | YP_008877641 | SPO0561 | ABC transporter |  | Sequence | SPO_PG036 | Confirmed by RNAseq | [42,43], This work |
| AHB86023 | YP_008877654 | SPO2024 | Aminotransferase |  | Sequence | SPO_PG038 |  | [42] |
| AHB86031 | YP_008877662 | SPO3316a | Stress protein |  | Sequence | SPO_PG039 |  | [42] |
| AHB86030 | YP_008877661 | SPO3904 | Heat shock protein |  | Sequence | SPO_PG037 | Confirmed by RNAseq | [42,43], This work |
| AAV95183 | YP_167141 | SPO1905 | Fumarate hydratase, class II | *fumC* | Sequence, ORF position |  |  | [42] |
| [AAV93547](http://www.ncbi.nlm.nih.gov/protein/AAV93547) | YP_165491 | SPO0222 | Alanine dehydrogenase | *ald* | Function |  |  | [24] |
| AAV93559 | YP_165503 | SPO0234 | Lysine dehydrogenase | *lysdh* | Function |  |  | [37] |
| AAV93560 | YP_165504 | SPO0235 | α-aminoadipic-δ-semialdehyde dehydrogenase | *aasadh* | Function |  |  | [37] |
| AAV93771 | YP_165716 | SPO0453 | DMSP lyase | *dddW* | Function |  |  | [26] |
| AAV93905 | YP_165850 | SPO0590 | LacI family transcriptional regulator | *hpsR* | Function |  |  | [29] |
| AAV93906 | YP_165851 | SPO0591 | Dihydroxypropanesulfonate (DHPS) TRAP transporter | *hpsK* | Function |  |  | [29] |
| AAV93907 | YP_165852 | SPO0592 | Dihydroxypropanesulfonate (DHPS) TRAP transporter | *hpsL* | Function |  |  | [29] |
| AAV93908 | YP_165853 | SPO0593 | Dihydroxypropanesulfonate (DHPS) TRAP transporter | *hpsM* | Function |  |  | [29] |
| AAV93909 | YP_165854 | SPO0594 | Dihydroxypropanesulfonate-3-dehydrogenase | *hpsN* | Function |  |  | [29] |
| AAV93910 | YP_165855 | SPO0595 | R or S-dihydroxypropanesulfonate-2-dehydrogenase | *hpsO* | Function |  |  | [29] |
| AAV93911 | YP_165856 | SPO0596 | S or R-dihydroxypropanesulfonate-2-dehydrogenase | *hpsP* | Function |  |  | [29] |
| AAV93912 | YP_165857 | SPO0597 | UspA stress protein | *hpsQ* | Function |  |  | [29] |
| AAV93913 | YP_165858 | SPO0598 | Membrane-bound sulfolactate dehydrogenase | *slcD* | Function |  |  | [33] |
| AAV93965 | YP_165912 | SPO0657 | Metallochaperone, putative | *naaT* | Function |  |  | [31] |
| AAV93966 | YP_165913 | SPO0658 | N-acetyltaurine amidohydrolase | *naaS* | Function |  |  | [31] |
| AAV93967 | YP_165914 | SPO0659 | LysR family transcriptional regulator | *naaR* | Function |  |  | [31] |
| AAV93968 | YP_165915 | SPO0660 | N-acetyltaurine ABC transporter, periplasmic binding protein | *naaA* | Function |  |  | [31] |
| AAV93969 | YP_165916 | SPO0661 | N-acetyltaurine ABC transporter, permease protein | *naaB* | Function |  |  | [31] |
| AAV93970 | YP_165917 | SPO0662 | N-acetyltaurine ABC transporter, permease protein | *naaB'* | Function |  |  | [31] |
| AAV93971 | YP_165918 | SPO0663 | N-acetyltaurine ABC transporter, ATP-binding protein | *naaC* | Function |  |  | [31] |
| AAV93972 | YP_165919 | SPO0664 | N-acetyltaurine ABC transporter, ATP-binding protein | *naaC'* | Function |  |  | [31] |
| AAV93981 | YP_165928 | SPO0673 | Taurine-pyruvate aminotransferase | *tpa* | Function |  |  | [24] |
| AAV93982 | YP_165929 | SPO0674 | Taurine ABC transporter, periplasmic taurine-binding protein | *tauA* | Function |  |  | [24] |
| [AAV93983](http://www.ncbi.nlm.nih.gov/protein/AAV93983) | YP_165930 | SPO0675 | Taurine ABC transporter, ATP-binding protein | *tauB* | Function |  |  | [24] |
| [AAV93984](http://www.ncbi.nlm.nih.gov/protein/AAV93984) | YP_165931 | SPO0676 | Taurine ABC transporter, permease protein | *tauC* | Function |  |  | [24] |
| AAV94086 | YP_166034 | SPO0781 | Phosphonate ABC transporter substrate-binding protein | *phnD* | Function |  |  | This work |
| AAV94436 | YP_166387 | SPO1136 | Diaminobutyric acid transaminase | *doeD* | Function |  |  | [40] |
| AAV94437 | [YP_166388](http://www.ncbi.nlm.nih.gov/protein/56696034) | SPO1137 | Aspartate-semialdehyde dehydrogenase | *doeC* | Function |  |  | [40] |
| AAV94438 | [YP_166389](http://www.ncbi.nlm.nih.gov/protein/56696035) | SPO1138 | AsnC/Lrp-like DNA-binding protein, transcriptional regulator | *doeX* | Function |  |  | [40] |
| AAV94439 | [YP_166390](http://www.ncbi.nlm.nih.gov/protein/56696036) | SPO1139 | Nα-acetyl-L-2,4-diaminobutyric acid deacetylase | *doeB* | Function |  |  | [39,40] |
| AAV94440 | [YP_166391](http://www.ncbi.nlm.nih.gov/protein/56696037) | SPO1140 | Ectoine hydrolase | *doeA* | Function |  |  | [39,40] |
| AAV94441 | YP_166392 | SPO1141 | Ectoine utilization protein EutC | *eutC* | Function |  |  | [39] |
| AAV94443 | YP_166394 | SPO1143 | Ectoine utilization protein EutA | *eutA* | Function |  |  | [39] |
| AAV94445 | YP_166396 | SPO1145 | Ectoine/5-hydroxyectoine TRAP transporter, periplasmic binding protein | *uehC* | Function |  |  | [39] |
| AAV94446 | YP_166397 | SPO1146 | Ectoine/5-hydroxyectoine TRAP transporter, small integral membrane protein | *uehB* | Function |  |  | [39] |
| AAV94447 | YP_166398 | SPO1147 | Ectoine/5-hydroxyectoine TRAP transporter, large integral membrane protein | *uehA* | Function |  |  | [39] |
| AAV94448 | YP_166399 | SPO1148 | Transcriptional regulator, GntR family | *gntR* | Function |  |  | [39] |
| AAV94838 | YP_166792 | SPO1551 | Trimethylamine (TMA) monooxygenase | *tmm* | Function |  |  | [45] |
| AAV94883 | YP_166837 | SPO1596 | DMSP lyase | *dddQ* | Function |  |  | [23] |
| AAV94987 | YP_166942 | SPO1703 | DMSP lyase | *dddD* | Function |  |  | [23] |
| AAV95191 | YP_167149 | SPO1914 | NADPH-dependent acrylyl-CoA reductase | *acuI* | Function |  |  | [27] |
| AAV95224 | YP_167183 | SPO1948 | Phosphate ABC transporter substrate-binding protein | *pstS* | Function |  |  | This work |
| AAV95316 | YP_167275 | SPO2045 | 3-methylmercaptopropionyl-CoA ligase | *dmdB* | Function |  |  | [2] |
| AAV95561 | YP_167522 | SPO2299 | DMSP lyase | *dddP* | Function |  |  | [23] |
| AAV95616 | YP_167578 | SPO2355 | Isethionate dissimilation regulator | *iseR* | Function |  |  | [25] |
| AAV95617 | YP_167579 | SPO2356 | Isethionate TRAP transporter | *iseM* | Function |  |  | [25] |
| AAV95618 | YP_167580 | SPO2357 | Isethionate TRAP transporter | *iseL* | Function |  |  | [25] |
| AAV95619 | YP_167581 | SPO2358 | Isethionate TRAP transporter | *iseK* | Function |  |  | [25] |
| AAV95620 | YP_167582 | SPO2359 | Isethionate dehydrogenase | *iseJ* | Function |  |  | [25] |
| AAV95731 | YP_167694 | SPO2477 | Manganese uptake regulator | *mur* | Function |  |  | [41] |
| AAV96422 | YP_168390 | SPO3187 | (2R)-3-sulfolactate dehydrogenase | *comC* | Function |  |  |  |
| AAV96534 | YP_168503 | SPO3307 | Lysine-ketoglutarate reductase | *lkr* | Function |  |  | [37] |
| AAV96590 | YP_168559 | SPO3363 | Manganese ABC transporter, permease protein | *sitD* | Function |  |  | [41] |
| AAV96591 | YP_168560 | SPO3364 | Manganese ABC transporter, permease protein | *sitC* | Function |  |  | [41] |
| AAV96592 | YP_168561 | SPO3365 | Manganese ABC transporter, ATP-binding protein | *sitB* | Function |  |  | [41] |
| AAV96593 | YP_168562 | SPO3366 | Manganese ABC transporter, periplasmic protein | *sitA* | Function |  |  | [41] |
| AAV96782 | YP_168752 | SPO3557 | Sulfite dehydrogenase subunit SoeC; transmembrane sulfate transporter | *soeC* | Function |  |  | [36] |
| AAV96783 | YP_168753 | SPO3558 | Sulfite dehydrogenase iron-sulfur cluster-binding subunit SoeB; cytosolic protein | *soeB* | Function |  |  | [36] |
| AAV96784 | YP_168754 | SPO3559 | Sulfite dehydrogenase molybdopterin cofactor-binding subunit SoeA; cytosolic protein | *soeA* | Function |  |  | [36] |
| [AAV96785](http://www.ncbi.nlm.nih.gov/protein/AAV96785) | YP_168755 | SPO3560 | Phosphate acetyltransferase | *pta* | Function |  |  | [24] |
| AAV96787 | YP_168757 | SPO3562 | Taurine transcriptional regulator | *tauR* | Function |  |  | [32] |
| AAV97018 | YP_168992 | SPO3804 | 3-methylmercaptopropionyl-CoA dehydrogenase | *dmdC* | Function |  |  | [2] |
| AAV97019 | YP_168993 | SPO3805 | Methylthioacryloyl-CoA hydratase | *dmdD* | Function |  |  | [2] |
| AAV97293 | YP_164988 | SPOA0157 | Sulfite exporter | *cuyZ* | Function |  |  | [30] |
| AAV97294 | YP_164989 | SPOA0158 | L-cysteate sulfo-lyase | *cuyA* | Function |  |  | [30] |
| AAV97295 | YP_164990 | SPOA0159 | Transcriptional regulator cuyR | *cuyR* | Function |  |  | [30] |
| [AAV96786](http://www.ncbi.nlm.nih.gov/protein/AAV96786) | YP_165136 | SPOA0309 | Sulphoacetaldehyde acetyltransferase | *Xsc* | Function |  |  | [24], This work |
| AHB86005 | YP_008877636 | SPO0344a | Hypothetical protein |  | New ORF | SPO_PG005 |  | [42] |
| AHB86006 | YP_008877637 | SPO0346a | Hypothetical protein |  | New ORF | SPO_PG006 |  | [42] |
| AHB86007 | YP_008877638 | SPO0360a | Hypothetical protein |  | New ORF | SPO_PG016 |  | [42] |
| AHB86008 | YP_008877639 | SPO0491a | Hypothetical protein |  | New ORF | SPO_PG018 |  | [42] |
| AHB86009 | YP_008877640 | SPO0504a | Hypothetical protein |  | New ORF | SPO_PG009 | Confirmed by RNAseq | [42,43], This work |
| AHB86011 | YP_008877642 | SPO0628a | Hypothetical protein |  | New ORF | SPO_PG013 | Confirmed by RNAseq | [42,43], This work |
| AHB86013 | YP_008877644 | SPO1044a | Hypothetical protein |  | New ORF | SPO_PG017 |  | [42] |
| AHB86014 | YP_008877645 | SPO1094a | Hypothetical protein |  | New ORF | SPO_PG022 | Confirmed by RNAseq | [42,43], This work |
| AHB86015 | YP_008877646 | SPO1226a | Hypothetical protein |  | New ORF | SPO_PG021 |  | [42] |
| AHB86016 | YP_008877647 | SPO1252a | Transcriptional regulator |  | New ORF | SPO_PG025 | Confirmed by RNAseq | [42,43], This work |
| AHB86017 | YP_008877648 | SPO1310a | Hypothetical protein |  | New ORF | SPO_PG011 |  | [42] |
| AHB86018 | YP_008877649 | SPO1337a | Hypothetical protein |  | New ORF | SPO_PG023 |  | [42] |
| AHB86019 | YP_008877650 | SPO1352a | Hypothetical protein |  | New ORF | SPO_PG003 |  | [42] |
| AHB86020 | YP_008877651 | SPO1356a | Signal transduction |  | New ORF | SPO_PG019 |  | [42] |
| AHB86021 | YP_008877652 | SPO1364a | Hypothetical protein |  | New ORF | SPO_PG008 |  | [42] |
| AHB86022 | YP_008877653 | SPO1412a | Hypothetical protein |  | New ORF | SPO_PG007 |  | [42] |
| AHB86024 | YP_008877655 | SPO2213a | Hypothetical protein |  | New ORF | SPO_PG010 |  | [42] |
| AHB86025 | YP_008877656 | SPO2341a | Hypothetical protein |  | New ORF | SPO_PG014 |  | [42] |
| AHB86026 | YP_008877657 | SPO2478 | RNA helicase |  | New ORF | SPO_PG024 |  | [42] |
| AHB86027 | YP_008877658 | SPO2652a | Polyketide cyclase |  | New ORF | SPO_PG020 |  | [42] |
| AHB86029 | YP_008877660 | SPO2973a | Hypothetical protein |  | New ORF | SPO_PG002 | Confirmed by RNAseq | [42,43], This work |
| AHB86032 | YP_008877663 | SPO3452a | Hypothetical protein |  | New ORF | SPO_PG015 |  | [42] |
| AHB86033 | YP_008877664 | SPO3498a | Hypothetical protein |  | New ORF | SPO_PG012 |  | [42] |
| AHB86034 | YP_008877665 | SPO3673a | Hypothetical protein |  | New ORF | SPO_PG026 | Confirmed by RNAseq | [42,43], This work |
| AHC32567 | - | SPOA0087a | Esterase-lipase |  | New ORF | SPOA_PG004 | Confirmed by RNAseq | [42,43], This work |
| AHC32568 | - | SPOA0272a | Hypothetical protein |  | New ORF | SPOA_PG001 | Confirmed by RNAseq | [42,43], This work |
| AAV93363 | YP_165305 | - | Hypothetical protein |  | Removed ORF | SPO0032 | Determined by RNAseq, Glimmer 3 | [43] This work |
| AAV93658 | YP_165605 | - | Hypothetical protein |  | Removed ORF | SPO0341 | Determined by RNAseq, Glimmer 3, ORF Finder | [43], This work |
| AAV94715 | YP_166669 | - | Hypothetical protein |  | Removed ORF | SPO1428 | Determined by RNAseq, Proteogenomics | [42,43], This work |
| AAV96892 | YP_168865 | - | Hypothetical protein |  | Removed ORF | SPO3670 | Determined by RNAseq, Glimmer 3 | [43], This work |
| AAV97542 | YP_165238 | - | Hypothetical protein |  | Removed ORF | SPOA0411 | Determined by RNAseq | [43], This work |

Reference only in Table S1:

45. Chen Y, Patel NA, Crombie A, Scrivens JH, Murrell JC. **Bacterial Flavin-Containing Monooxygenase Is Trimethylamine Monooxygenase**. *Proc Natl Acad Sci USA.* 2011; 108:17791–96. PubMed http://dx.doi.org/10.1073/pnas.1112928108.
